# A decision analysis of long-term lithium treatment and the risk of renal failure

**DOI:** 10.1111/j.1600-0447.2012.01847.x

**Published:** 2012-09

**Authors:** U Werneke, M Ott, E Salander Renberg, D Taylor, B Stegmayr

**Affiliations:** 1 Division of Psychiatry, Department of Clinical Sciences, Umeå UniversityUmeå, Sweden; 2 Division of Internal Medicine, Department of Nephrology, Sunderby HospitalLuleå, Sweden; 3 Pharmacy Department, South London and Maudsley NHS Foundation Trust, Institute of Pharmaceutical Sciences, King's CollegeLondon, UK; 4 Institute of Public Health and Clinical Medicine, Umeå UniversityUmeå, Sweden

**Keywords:** bipolar disorder, decision analysis, end-stage renal disease, lithium, suicide

## Abstract

**Objective:**

To establish whether lithium or anticonvulsant should be used for maintenance treatment for bipolar affective disorder (BPAD) if the risks of suicide and relapse were traded off against the risk of end-stage renal disease (ESRD).

**Method:**

Decision analysis based on a systematic literature review with two main decisions: (1) use of lithium or at treatment initiation and (2) the potential discontinuation of lithium in patients with chronic kidney disease (CKD) after 20 years of lithium treatment. The final endpoint was 30 years of treatment with five outcomes to consider: death from suicide, alive with stable or unstable BPAD, alive with or without ESRD.

**Results:**

At the start of treatment, the model identified lithium as the treatment of choice. The risks of developing CKD or ESRD were not relevant at the starting point. Twenty years into treatment, lithium still remained treatment of choice. If CKD had occurred at this point, stopping lithium would only be an option if the likelihood of progression to ESRD exceeded 41.3% or if anticonvulsants always outperformed lithium regarding relapse prevention.

**Conclusion:**

At the current state of knowledge, lithium initiation and continuation even in the presence of long-term adverse renal effects should be recommended in most cases.

**Significant outcomes**

- At the current state of knowledge, lithium initiation and even continuation in the presence of long-term adverse renal effects should be recommended in most cases.
- Psychiatrists should not withhold or discontinue effective treatments for fear of somatic adverse effects.
- A nephrologist referral should in most cases concern the management of the associated risks of continued lithium use rather than the decision of whether to stop lithium.

**Limitations**

- Like any statistical model, our decision analysis will not capture the full complexity of the clinical decision making in individual patients.
- The validity of our model is limited by the quality of the data feeding the model. To account for the uncertainties of our estimates derived from the literature, we conducted a sensitivity analysis.
- There was not sufficient evidence to include second-generation antipsychotics. However, the effects of these on both branches of our decision tree would most likely have canceled out.

## Introduction

Lithium remains a first-line treatment for maintenance treatment in patients with bipolar affective disorder (BPAD). However, its use seems in decline, with anticonvulsants (ACs) and some second-generation antipsychotics (SGAs) being increasingly used as alternatives despite a much more limited evidence base (1). It is unclear why this trend away from lithium has occurred. Potential reasons may include lack of training of psychiatrists in its use and ‘aggressive marketing of alternative medication that are patentable and therefore more profitable’ (1). Even today, 60 years after its debut (2), the mechanism of the mood-stabilizing action of lithium remains largely unexplained. This rests uncomfortably with those demanding a treatment rationale going beyond empiricism (3) although the mechanism of action for ACs also remains unknown.

The narrow therapeutic index of lithium and the potential for serious adverse effects is another concern. The debate about the risk of kidney damage and the risks and benefits of lithium treatment began soon after licensing, and its use has remained controversial. In 1981, Schou and Vestergaard asked ‘Are we buying the mental health of lithium treatment at the expense of their kidney function and survival? Should we perhaps stop using lithium? Should we avoid using it for a period longer than a few years?’ (4) Long-term studies have emerged showing that lithium can significantly impair renal function. Lithium can affect renal function in two ways. Tubular damage leading to polyuria and diabetes insipidus renalis is relatively common and occurs early during treatment. It may become irreversible in 15% of patients after long-term lithium exposure (5). Glomerular damage affecting the renal filtration and clearance ability is rarer and emerges late, often after decades of treatment. It remains unclear how many such patients who develop chronic kidney disease (CKD) as a consequence of glomerular damage progress to end-stage renal disease (ESRD), that is to such a severe impairment of the kidney function that dialysis is required. Monitoring renal glomerular function regularly has become standard in lithium maintenance therapy. The most recent long-term study available emphasized that ESRD, albeit uncommon, was not rare and ‘more prevalent than previously thought’(6). In addition, lithium is not recommended for use in patients with severe renal impairment (7). This has resulted in an uncertainty about whether to recommend lithium at all and whether to continue lithium once CKD has occurred.

Equally, it remains unclear whether seeking expert advice on renal changes leads to better clinical outcomes because nephrologists are basically faced with the same dilemma as psychiatrists. Would switching to another mood stabilizer at such a late stage increase the risk of suicide or relapse and if so would the risk be worth taking to preserve kidney function? After all, only few such patients seem to progress to ESRD and lithium discontinuation does not guarantee renal recovery. Moreover, other mood stabilizers are also associated with significant adverse effects such as weight gain and diabetes mellitus. The scientific literature gives very little guidance in this question as trials and meta-analyses are not powered to quantify serious but uncommon adverse effects. A decision analysis lends itself as a method to address this question because treatment effects and risks can be considered at the same time.

### Aims of the study

To establish whether, based on the current state of knowledge, lithium or anticonvulsants should be used for maintenance treatment for bipolar affective disorder if the risks of suicide and relapse were traded off against the risk of end-stage renal disease.

## Material and methods

We conducted a decision analysis simulating the real-world decision process between physicians and patients in the consulting room comparing the relative risks and utilities of two mood stabilizers for the maintenance treatment for BPAD. The analysis addressed two questions relevant to prescribers: i) Should lithium be recommended at the beginning of treatment in view of a small but significant risk of ESRD later in life? and ii) Should lithium continuation be recommended even in the presence of long-term adverse renal effects?

This involved weighing up the need for effective relapse and suicide prevention right from the beginning of treatment, with the risk of lithium-associated ESRD occurring many years later. It was assumed that if signs of renal impairment emerged, physicians would have to reconsider the use of lithium.

### Structuring the decision process

The decision of whether to take lithium was the starting point of the analysis. Once a diagnosis of bipolar affective or a related disorder is established, the question of which mood stabilizer to use as maintenance therapy arises. Should the patient commence on lithium or rather on other mood stabilizers such as ACs? The clinical decision is a trade-off between the expected effects and side-effects, in this case between relapse and suicide prevention as the desired effect and ESRD as the most undesirable side-effect. To structure this problem, we used the decision software TreeAge Pro2009 and 2011 (8).

### The model

The decision analysis uses a tree, each branch representing two options. A square node represents a decision made by the clinician and/or patient and circular nodes present chance events beyond the control of the decision-makers. There were two main decisions to take. The first decision concerns the use of lithium versus ACs at treatment initiation weighing up the risk of suicide with the probability of developing CKD. The second decision concerns the potential discontinuation of lithium in patients displaying signs of CKD typically after 20 years of lithium treatment. In our model, this decision depends on the risk of suicide and the likelihood to develop ESRD continuing or discontinuing lithium. The final endpoint of the analysis was 30 years of treatment with five potential outcomes to consider: death from suicide, unstable BPAD and ESRD, unstable BPAD but no ESRD, stable BPAD but ESRD, and stable BPAD and no ESRD. We did not factor SGAs into our model because we could not identify sufficient data to populate the model. Also it is much more likely at present that SGAs are added on as a second mood stabilizer to existing treatment, which effectively removes them from the equation in our model.

### Estimating probabilities and valuing outcomes

To populate the tree, we derived the probabilities for the different chance nodes (Table 1) from a systematic literature review. The sum of all probabilities of the options at a respective chance node had always to be one. For all nodes, we conducted a sensitivity analysis considering worse and best case scenarios derived either from different results published in the literature or from 95% confidence intervals around our baseline assumptions.

**Table 1:** Assumptions for the decision tree

Parameters (variable name)	Baseline probability	Range	References	Comment
*Risk of suicide*				
Cumulative suicide risk with lithium in the first 20 years of treatment until the second decision point is reached	1.6%	0.6–12.8%	9–13	Linear decline in the first 30 years of treatment
Cumulative suicide risk with lithium in the last 10 years of treatment	0.8%	0.3–6.4%	9–13	Linear decline in the first 30 years of treatment
Cumulative suicide risk with AC in the first 20 years of treatment	8.1%	0.6–12.8%	9, 10, 12, 13	Linear decline in the first 30 years of treatment
Cumulative suicide risk with AC in the last 10 years of treatment	4.1%	0.3–6.4%	9, 10, 12, 13	Linear decline in the first 30 years of treatment
Suicide risk within the next 10 years if switching from lithium to AC	4.1%	0.6–8.4%	9, 13, 14, 15	Range: no risk increase of suicide risk doubling of suicide risk
*Risk of unstable BPAD*				
Risk of unstable BPAD with lithium treatment	59% overall, 58.2% excluding suicides	20–74%	16–21	All suicides occur in pts with unstable BPAD. Range: includes the possibility that AC is superior to lithium
Risk of unstable BPAD with AC treatment	69% overall, 64,9% excluding suicides	23–74%	16–19, 21	All suicides occur in pts with unstable BPAD. Range: includes the possibility that lithium is superior to AC
Risk of unstable BPAD after switch to ACs after 20 years of lithium treatment	69% overall, 64,9% excluding suicides	23–74%		As for AC treatment
*Risk of renal impairment*				
Risk CKD after 20 years of lithium treatment: S-Cr ≥150 μm	4.3.%	1.7–41.1%	6, 22	Assumption that all cases of CKD occurred in pts taking lithium long term
Risk of ESRD in the general population	0.08%	0.04–1.2%	6	Best and worst case scenario: 95% CI of baseline estimate
Risk of ESRD in patients with CKD continuing lithium	14.0%	0.08–37.5%	6, 23	Range: no progression and ESRD risk as baseline population – substantial deterioration of CKD
Risk of ESRD in patients with CKD discontinuing lithium	14.0%	0.08–37.5%	6, 23	Range: full recovery of kidneys – substantial deterioration of CKD
*Utilities for the different endpoints*				
Suicide	0.00			
Renal replacement therapy	0.62	0.44–0.86	25, 26	Best case: renal transplant; worst case: long term hemodialysis
Stable BPAD and no ESRD	0.80	0.38–0.86	27–32	
Stable BPAD but ESRD	0.50	0.17–0.74	27–32	Utility score renal replacement therapy x utility score stable BPAD
Unstable BPAD but no ESRD	0.34	0.12–0.76	27–32	Considering manic and depressive relapses. Worst case: inpatient mania, best case: outpatient depression
Unstable BPAD and ESRD	0.21	0.07–0.47	27–32	Utility score renal replacement therapy x utility score unstable BPAD

AC, anticonvulsants; BPAD, bipolar affective disorder; CI, confidence interval; CKD, chronic kidney disease; ESRD, end-stage renal disease.

We based our assumptions about the risk of suicide while undergoing treatment with lithium or ACs on two meta-analyses conducted by Baldessarini & Tondo (9) and Baldessarini et al. (10). To assess the long-term suicide risk, we relied on the cohort study conducted by Angst et al. (11) capturing the clinical course of patients with BPAD over 40 years. From the annual suicide risk estimates obtained, we modeled the cumulative suicide risk within the first 20 years on a hypothetical cohort. To obtain the subsequent cumulative suicide risk from year 21 to 30, we multiplied the suicide risk within 20 years with 0.5 because the risk remained linear in the first 30 years and only thereafter leveled out. The best and worst case scenarios were the same for ACs and lithium, assuming that both drugs would perform equally at both extremes. The range covered estimates from a meta-analysis by Cipriani et al. (12) and a large retrospective cohort study comparing lithium and valproate by Goodwin et al. (13). Regarding suicide risk after switching from lithium to ACs after 20 years, we applied our baseline assumption for ACs. We also considered a large register study, which showed that switching to ACs from lithium would not affect the suicide risk whereas switching from ACs to lithium would lead to a reduction of risk (14). For our model, we inferred that the suicide risk would remain unchanged in the best case (9, 13) and double in the worst case scenario (14, 15).

We obtained our assumptions about the risk of relapse from the BALANCE trial, which is a direct comparison of lithium and valproate monotherapy (16), and the DUAG-6 trial, which is a direct comparison of lithium and lamotrigine monotherapy (17). We validated the assumptions derived from these trials with the findings from the two most recent large register studies comparing lithium with valproate on the one hand (18) and lithium with lamotrigine on the other (19). We defined the risk of unstable BPAD as a likelihood of a yearly risk of relapse ≥0.5 corresponding to an episode free interval of at least 6 months. The nature of the decision tree demanded choosing only one value per chance node as the baseline assumption. For this, we pragmatically chose the findings from the BALANCE study (16) as this was the largest trial available. The relapse rates from the other three studies largely corresponded. Specifically, the results of the two register studies also indicated superiority of lithium to ACs (18, 19). The DUAG-6 trial did not demonstrate any significant difference in effectiveness between lithium and lamotrigine (17). However, the findings of the DUAG-6 trial lay comfortably in the range of the sensitivity analysis, which provided for the possibility of equality or even superiority of maintenance treatment with ACs.

For the sensitivity analysis, we based our best case scenario on the risk of episodes leading to admission to hospital only according to the BALANCE estimates (16) and the worst case scenario on the likelihood of ten years freedom from relapse (20). This range not only covered the estimates from most recent register-based cohort study (18, 19) but also the meta-analysis by Beynon et al. (21). We used the same assumptions regarding the risk of relapse for the first decision point at treatment initiation and for the second decision point after 20 years of treatment.

We derived our baseline and best/worst case assumptions for lithium-associated CKD and risk of subsequent ESRD if lithium was continued or discontinued from the long-term cross-sectional studies conducted by Bendz et al. (6) and Bassilios et al. (22) as well as a case–control study by Lepkifker et al. (23). The most recent study (6), covering two Swedish counties identified 1313 patients treated with lithium ≥15 years and 59 patients with CKD or ESRD. Two of the patients developing ESRD had not had CKD as defined as serum creatinine ≥150 μmol (1.7 mg/dl) at lithium discontinuation. This would yield a CKD prevalence of 4.3% for our model (57/1313) under the assumption that CKD occurred after at least 15 years of treatment. Of 18 patients with ESRD, eight were continuing lithium while ten progressed to ESRD despite lithium discontinuation. Thus, although the study did not report in how many patients CKD was successfully halted after lithium discontinuation, we assumed that of the 57 patients with CKD an equal proportion of those who had continued and discontinued lithium progressed to ESRD. Others have reported similar results (24).

We valued outcomes using utilities as a measure of patient preference on a scale from zero to one with one denoting a perfect state of health. The utility of having committed suicide was zero. For the other endpoints, we derived the utility scores for hemodialysis, renal transplantation (25, 26), and stable/unstable BPAD from the relevant literature (27–32). As we could not find any utility data for a combined state of BPAD and ESRD, we multiplied the individual utility scores.

### Calculating the tree

We then calculated the tree to obtain a numerical estimate for the best strategy to choose. This involved folding (rolling) back the tree starting with the outcomes and working through the tree in a backwards fashion, that is from right to left, until arriving at the index decision. The utility of each outcome with the assigned probability was multiplied and summed up. The obtained result would then represent the value of the next chance node down, and the procedure was repeated for the next chance node.

### Sensitivity analysis

In the final step, we conducted a sensitivity analysis to evaluate the stability of our conclusions by varying the probabilities of the factors featured at chance nodes. For each node, we had formulated best and worst case scenarios and used a one-way sensitivity analysis to evaluate the expected utility across that range varying one factor at the time holding all other factors stable. We also tested the range of utilities keeping the chance nodes stable. We used a one-way sensitivity analysis to determine the thresholds or break-even points where either decision would be a ‘toss-up’, that is, the outcomes in terms of expected utilities would be the same (33). Beyond that threshold, the decision strategy would change to the alternative treatment. For the second decision point, we applied two-way sensitivity analysis to examine the impact of simultaneous changes in two variables. Here, we analyzed the impact of the simultaneous changes in the likelihoods to develop ESRD continuing or discontinuing lithium. We also modeled the trade-offs for the risk of developing ESRD continuing lithium against the risk of suicide or relapse of BPAD while treated with lithium. In a further two-way sensitivity analysis, we modeled the trade-offs for the risk of developing ESRD discontinuing lithium against the risk of suicide or relapse of BPAD if switching to ACs. We then conducted a three-way sensitivity analysis trading off the risk of developing ESRD against the risks of suicide and unstable BPAD at the same time. Varying the three variables over their estimated range at the same time over 10 levels yielded 11 data points for each of the three variables involved. Thus, 1331 scenarios were calculated. From these we confirmed the treatment recommendation for the scenario using the baseline assumptions and calculated which proportion of scenarios would recommend one strategy over the other. Finally, we identified the most critical assumptions in our decision analysis indicating future research priorities by creating a tornado diagram (named after its shape). This diagram demonstrates and ranks the degree to which uncertainty at individual variables affects incremental utility, that is, change of utility from the baseline value between the two strategies; the wider a bar, the larger the uncertainty. Deviations to the right showed potential gains in utility. From the diagram, we derived ‘risk per cent’ as a measure of spread, indicating how much each specified variable would contribute to the total uncertainty (8).

## Results

**Fig. 1:**
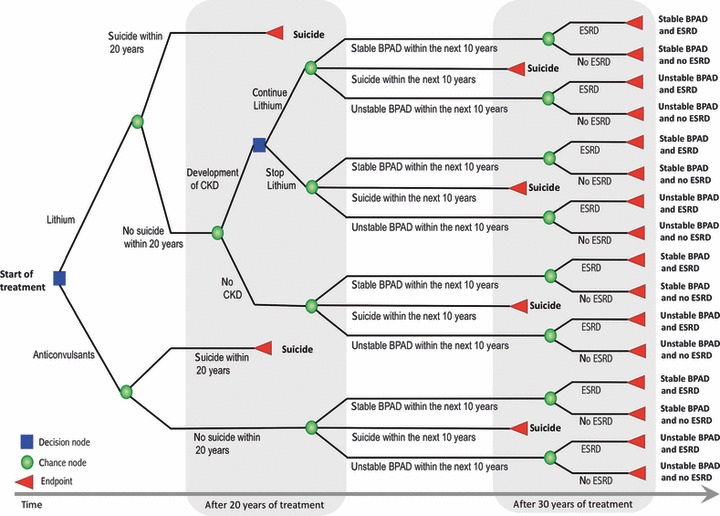
Decision tree. CKD, chronic kidney disease; ESRD, end stage renal disease; BPAD, bipolar affective disorder.

The baseline model relied on the assumptions derived from the literature outlined in Table 1 and recommends choosing lithium over ACs at both decision points, that is, at start of treatment and 20 years into treatment. Folding back the tree yielded an expected utility at the start of treatment of 0.50 for lithium and of 0.42 for ACs (decision point 1). In patients having developed CKD after 20 years of lithium treatment, the tree yielded an expected utility of 0.50 for lithium continuation and of 0.44 for switching to ACs (decision point 2) (Fig. 1).

### Decision point 1: lithium vs. ACs at the beginning of treatment

**Fig. 2:**
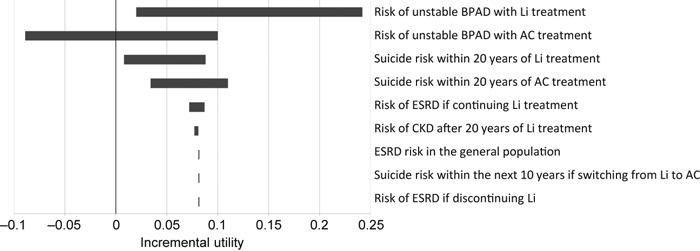
Tornado Diagram at lithium vs. anticonvulsants. BPAD, bipolar affective disorder; Li, lithium; AC, anticonvulsants; SUI, suicide; ESRD, end stage renal disease; CKD, chronic kidney disease.

At start of treatment, concerns over mental health were the sole driver for the treatment decision. Varying the risk of suicide or the risk of progressing to CKD or ESRD over the assumed range did not change the treatment recommendation at decision point 1. However, if the risk of unstable BPAD would be 47.4% or less in ACs treatment, the model would recommend ACs instead of lithium. The risk of unstable BPAD with either lithium or ACs accounted for 87.7% of the total uncertainty in the model and the risk of suicide for a further 12.4%. The uncertainty about the risk CKD and ESRD only played a negligible role at the initial treatment decision (Fig. 2).

### Decision point 2: continue or discontinue lithium after 20 years of treatment once CKD has occurred

**Fig. 3:**
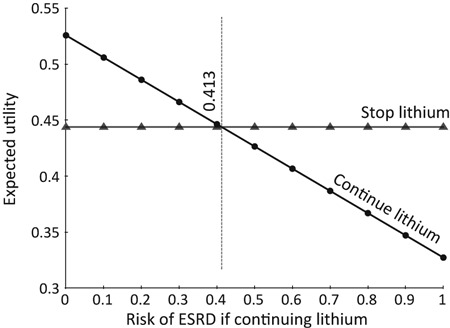
One-way sensitivity analysis on risk of end stage renal disease if continuing lithium.

Twenty years into the treatment, however, the perspective changed. Now, some patients would have developed CKD. Some of these would progress to ESRD. At that point, the decision was about whether to continue or stop lithium and switch to ACs. The model recommended a switch from lithium to ACs at this point if the risk of unstable BPAD was more than 71.4% with lithium treatment or less than 56.6% with AC treatment. Varying the risk of suicide did not affect the treatment recommendation. Neither did the risk of progression to CKD or ESRD over the assumed range lead to a switch of strategy. To see how high the risk of progression to ESRD under lithium treatment would have to be to warrant a switch to ACs, we extended the range of the sensitivity analysis and found a threshold value of 41.3%, beyond which the model advised to change the strategy (Fig. 3).

We then examined further how the risks of suicide and relapse on the one hand and the risk of progression to ESRD on the other hand should be balanced by simultaneously changing these variables (Table 2). This two- and three-way analysis showed that the model opted for lithium for all of the baseline scenarios. In the two-way analysis, we first traded off the risk of ESRD continuing lithium against the risk of ESRD discontinuing lithium and kept the risk of suicide and relapse stable. This showed that in 97.3% of cases, lithium remained the treatment of choice. Most scenarios in which lithium was continued also endorsed lithium as the treatment of choice. If lithium was discontinued and the risk of ESRD was only traded off against the risk of suicide, the model pointed toward lithium in all cases. If the risk of relapse was considered at the same time, the model recommended switching to ACs in 59.9% of all the simulated scenarios. However, inspection of the individual scenarios showed that ACs would essentially have to outperform lithium regarding relapse prevention is such cases.

**Table 2:** Sensitivity analysis: Patients who have developed CKD after 20 years of lithium treatment – recommended strategies varying two or three variables at the same time

Clinical trade-offs of risks involved in the clinical decision	Variable 1	Variable 2	Variable 3	Baseline scenario – recommended strategy	All possible scenarios in the probability range – recommended strategy
ESRD with lithium vs. ESRD without lithium	Risk of ESRD in patients with CKD continuing lithium	Risk of ESRD in patients with CKD discontinuing lithium		Continue lithium	Continue lithium in 97.3% of cases
*Continuing lithium*					
ESRD vs. suicide	Risk of ESRD in patients with CKD continuing lithium	Risk of SUI within the next 10 years		Continue lithium	Continue lithium in 98.2% of all cases
ESRD vs. relapse	Risk of ESRD in patients with CKD continuing lithium	Risk of unstable BPAD with lithium treatment		Continue lithium	Continue lithium in 90.7% of all cases
ESRD vs. suicide or relapse*	Risk of ESRD in patients with CKD continuing lithium	Risk of suicide within the next 10 years	Risk of unstable BPAD with lithium treatment	Continue lithium	Continue lithium in 84.4% of all cases
*Discontinuing lithium*					
ESRD vs. suicide	Risk of ESRD in patients with CKD discontinuing lithium	Risk of suicide within the next 10 years after switching to AC		Continue lithium	Continue lithium in 100% of all cases
ESRD vs. relapse	Risk of ESRD in patients with CKD discontinuing lithium	Risk of unstable BPAD with AC treatment		Continue lithium	Switch to AC in 60.5% of all cases
ESRD vs. suicide or relapse*	Risk of ESRD in patients with CKD discontinuing lithium	Risk of suicide within the next 10 years after switching to AC	Risk of unstable BPAD with AC treatment	Continue lithium	Switch to AC in 59.9% of all cases

AC, anticonvulsant; BPAD, bipolar affective disorder; CKD, chronic kidney disease; ESRD, end-stage renal disease.

* Based on an animation of 1331 scenarios, that is, 10 intervals for each of the three variables.

**Fig. 4:**
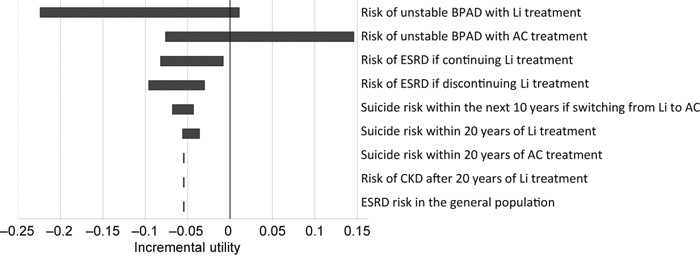
Tornado Diagram at continue lithium vs. stop lithium. BPAD, bipolar affective disorder; Li, lithium; AC, anticonvulsants; ESRD, end stage renal disease; SUI, suicide; CKD, chronic kidney disease.

Even at this decision point, the risk of unstable BPAD with either lithium or ACs was the most important variable in the model accounting for 90.6% of the total uncertainty. The risk of ESRD accounted for 8.6% and the risk of suicide for 0.8% (Fig. 4).

## Discussion

The findings of our decision suggest that at the current state of knowledge, lithium continuation should be recommended in most cases even in the presence of long-term adverse renal effects. In some patients, switching to ACs might become an option but only if ACs outperformed lithium regarding relapse prevention. Our decision analysis focused on outcomes at discrete points of time. Modeling of transitional states in between, although theoretically possible, would not have enhanced the accuracy of the model because of the lack of reliable data. Also, this would have made the model less transparent and clinically intuitive, and the ultimate goal of this model was to map the decision process as it takes place in the consulting room every day. At the starting point of maintenance treatment, concerns about relapse and – to a lesser extent – suicide prevention are most important eclipsing any considerations about ESRD in the remote future. After 20 years of treatment, our model indicates that the risk of relapse is still the main driver for the choice of a mood stabilizer even if CKD has occurred. Concerns about ESRD become more significant though. Concerns about suicide risk statistically play a subordinate role, presumably because the risk is much smaller than the risk of relapse. At this point, many patients are referred to a renal specialist. But the remit for such referrals may be unclear if the referring psychiatrist does not include a risk assessment, which can guide the renal specialist in his deliberations on whether to continue lithium or not. Specifically, the referrer should outline the expected consequences of discontinuing lithium and switching to ACs. The BALANCE study suggests that patients may fare better switching to combination therapy rather opting for valproate monotherapy (16). But this would not remove lithium from the equation. Thus, a nephrologist referral might better concern the management of the associated risks of continued lithium use in patients who have developed CKD such as cardiovascular problems, anemia, and mineral bone disease. Indeed, very little is known about the risk of suicide in patients switching from lithium to ACs at a late stage but it may be lower than previously thought (10). Patients who have not committed suicide within the first twenty years of BPAD may have a more benign course irrespective of the mood stabilizer chosen. But not all patients treated with lithium develop CKD and not all patients with CKD develop ESRD. Hence, there may be specific risk factors yet to be identified. Intuitively, it would appear that the risk of ESRD might be higher in those patients who continued lithium despite CKD. Clinical intuition would also suggest that lithium discontinuation would halt progression of CKD. However, this has not been shown (34) and it is now known that some patients have indeed progressed to ESRD despite lithium discontinuation (6, 24, 35). In our model, we relied on the only long-term population-based study which reported that half of all ESRD patients had progressed to this state despite having stopped lithium (6). Possibly, there is a ‘point of no return’ where CKD progresses irrespectively, and this threshold may lie between a creatinine clearance of 25 and 40 ml/min (24).

Like any statistical model, our decision analysis has some limitations, which may affect its generalizability and validity. In our decision analysis, in line with the BALANCE trial (16), we have not considered SGAs although they are increasingly used in the treatment for BPAD. Olanzapine and quetiapine have been licensed for the maintenance treatment for BPAD but only fairly recently so and we could not identify sufficient SGA data for inclusion into our decision tree. To our knowledge, there is only one study available exploring the risk of suicide while on olanzapine maintenance treatment (36) and trials assessing SGAs for maintenance monotherapy are not available at all. Currently, it also appears much more likely that SGAs are used in combination with other mood stabilizers so that the effect in both branches of the tree would have canceled out in any event. At present, it remains unclear whether maintenance treatment with SGAs is really tenable long-term. Physicians may switch to other mood stabilizers once adverse effects such as weight gain and impaired metabolic control emerge and the risk of cerebrovascular events increases (37). However, as demonstrated in the BALANCE trial (16), adding a second mood stabilizer to lithium may yield better results than switching to another agent. Again, this trial did not purport to address the risk of adverse effects that may potentiate with the length of treatment.

In our model, we did not consider the risk of diabetes insipidus although this is much more common than ESRD. However, this reflects recommended clinical practice where monitoring for glomerular renal impairment via serum creatinine or estimated glomerular filtration rate (eGFR) is routine but screening for tubular damage is uncommon (38). If the risk of ESRD was deemed clinically irrelevant, although rare, this would indeed compromise the validity of our model. But it would also imply that regular serum creatinine or eGFR monitoring should be abandoned. Also, ESRD as a long-term adverse effect may become more relevant in future because the first generation of patients who have received lithium maintenance therapy is coming off age (39). There is obviously a level of uncertainty around the assumptions made in our model. We addressed this by conducting a sensitivity analysis choosing generous ranges for the best and worst case scenarios covering the various estimates obtained from the literature review. For instance, there is still considerable uncertainty about the comparative effectiveness of lithium and ACs in long-term maintenance treatment for BPAD. Meta-analyses have too short a horizon to address this question. However, we based our assumptions about relapse prevention on the most recent trial and register data available (16–19). The only detailed long-term cohort study in the area does not include treatment with ACs (11). Regarding the risk of completed suicide, data were too limited to consider valproate on its own in the model, and the range of the sensitivity analysis covered the existing risk estimates. Some studies such as the DUAG-6 (17) study, the STEP-BD study (40), some other cohort studies (41, 42), and a recent meta-analysis (21) seem to point toward equivalence between lithium and ACs. Another recent trial of 98 patients with BPAD and past suicide attempts comparing lithium and valproate failed to detect a significant treatment difference regarding further suicide attempts (43). Thus, if lithium was ultimately to lose its status as therapeutically superior, adverse effects of other mood stabilizers might become more prominent in clinical decision making. Our model identifies this as an area of continued uncertainty and a research priority.

We did not conduct an economic analysis with this model because this would have shifted the focus to the likelihood of recurrences of manic and depressive episodes and the associated treatment costs. Such models already exist (44, 45) but tend not to factor in the costs of long-term physical adverse effects. Including costs in our model might have come to an inappropriate inverse conclusion because lithium treatment most likely increases life expectancy and renal replacement therapy is extremely costly.

The decision of whether to stop lithium treatment when faced with the risk of ESRD and the prospect of renal replacement therapy remains a huge clinical dilemma. Maintenance treatment for BPAD is a staple of psychiatric practice, yet the evidence on which clinicians and patients can base their treatment choices is extremely limited.

Indeed many patients taking mood stabilizers long-term may not adhere to treatment, and this adversely affect the clinical outcome. About 50% of patients taking lithium seem to discontinue their treatment within the first six months (46, 47), and others only take lithium intermittently (48). Regarding ACs, data on adherence to long-term treatment are not available. Failure to adhere is most likely multifactorial. Patient education, prevention of alcohol and substance abuse, and family involvement may improve adherence rates. Equally important in this context is the prevention and containment of side-effects (49).

Clearly, lithium is not only associated with renal adverse effects but also with other clinically relevant side- effects. Some notable side-effects such as psoriasis and hyperparathyroidism are less common with a prevalence of approximately 6% each (50–52). Other adverse effects such as hypothyroidism are more common. Persistent hypothyroidism may affect approximately 10% of lithium-treated patients but the prevalence may substantially rise in the elderly (48, 53, 54). Currently, we do not know how such adverse effects may affect the risk of relapse. A recent subanalysis of clinical trial data suggested that such physical adverse events may indeed be relevant to the mental status. Subjects with BPAD treated with lithium who required an intervention for a depressive episode had significantly higher levels of thyroid stimulating hormone (TSH) than those who did not (55). Neither are ACs side-effect free. Valproate carries a substantial risk of congenital malformations including an increased the risk of cardiac and neural tube defects. The risk is dose dependent and may exceed 20% at exposure to high doses (56). This limits the use of valproate maintenance treatment in women of childbearing age. Very rarely, valproate is associated with severe hepatotoxicity and pancreatitis but the true prevalence may be underestimated (57, 58). Lamotrigine is commonly associated with cutaneous reactions; about 10% of exposed patients develop some form of rash. Careful titration to the target dose decreases the risk. As the risk of rash seems dependent on the speed of titration rather than the absolute dose, it is possible to re-expose patients who have developed a rash using a lower speed of titration. However, about 0.1–0.3% of patients treated with lamotrigine develop serious rashes such as Steven Johnson syndrome (59). Unfortunately, in clinical practice it is not always easy to draw a clear distinction between ‘harmless’ and serious rashes (59). This uncertainty raises again a clinical dilemma if and when to discontinue or re-challenge in those patients who have responded well to lamotrigine but have developed a rash of ambiguous severity. Ultimately, irrespective of the treatment used, it is always important to find an acceptable trade-off between numbers needed to treat and numbers needed to harm (60).

Regarding long-term treatment for BPAD, our decision analysis is a first attempt to quantify the trade-off between effects and risks exploring the gray zone of the ‘reality in between’ (61). In common with previous findings (62), our study demonstrates that psychiatrists should not withhold or discontinue effective treatments for fear of somatic adverse effects. At the current state of knowledge, lithium initiation and even continuation in the presence of long-term adverse renal effects should be recommended in most cases. However, each case is different and we hope that our decision analysis provides support to clinicians who have to make difficult clinical decisions every day, particularly for those ‘close-call situations’ where the choice is not intuitively clear.
